# Structuring ontologies from natural language for collaborative scenario modeling in agri-food systems

**DOI:** 10.3389/frai.2022.1056989

**Published:** 2022-12-22

**Authors:** Romy Lynn Chaib, Catherine Macombe, Rallou Thomopoulos

**Affiliations:** ^1^ITAP, INRAE, Institut Agro, Montpellier, France; ^2^IATE, University of Montpellier, INRAE, Institut Agro, Montpellier, France

**Keywords:** ontology, multicriteria argumentation, prospective, collaborative modeling, Godet method, MyChoice software

## Abstract

Prospective studies require discussing and collaborating with the stakeholders to create scenarios of the possible evolution of the studied value-chain. However, stakeholders do not always use the same words when referring to one idea. Constructing an ontology and homogenizing vocabularies is thus crucial to identify key variables, which serve in the construction of the needed scenarios. Nevertheless, it is a very complex and time-consuming task. In this paper we present the method we used to manually build ontologies adapted to the needs of two complementary system-analysis models (namely the “Godet” and the “MyChoice” models), starting from interviews of the agri-food system's stakeholders. The objective of the paper is to explore whether and how prospective studies may have to gain from complementing the methodologies used (here Godet) with formal approaches from other disciplines, such as knowledge engineering (here MyChoice), which is usually not the case currently.

## 1. Introduction: A context of collaborative modeling

Ontologies represent the concepts used by people as well as the relationships between them (Uschold and Gruninger, 1996). They are very important when it comes to structuring knowledge from texts (Maedche and Staab, 2000), especially since they introduce standards allowing the use of formalized information and vocabularies in various studies (Nebot and Berlanga, 2009).

Using the same words to refer to one concept is essential when dealing with large and complex knowledge resources, such as agri-food value-chains. Those are complex systems, made of several stakeholders interacting with each other and with their environment (Croitoru et al., 2016). Those stakeholders can be primary matter producers, breeders, transformers, distributors, consumers, but also public and private institutions, researchers, technical centers, etc. All of them have different opinions as well as divergent priorities (Handayati et al., 2015), whether it is because of their position and implication in the value-chain, their political engagements, their affiliations or their life experience. They also have various different possible ways of saying the same thing: they use different ontologies to refer to same concepts. When it comes to taking a decision concerning the value-chain, it is best we have the vision and contribution of as many different points of view as possible, thus an implication of as many types of stakeholders as possible (Mitchell et al., 1997), which does not simplify the construction of a common ontology.

The case study on which this paper is based is the prospective study done on the French pork value-chain as part of project Sentinel. Indeed, the purpose of this French National Research Agency project is to improve food chemical safety along the value-chain by introducing new screening tools. In order to ensure durable applications of those tools, their impact on the value-chain must be anticipated. Nevertheless, to be able to assess the impacts of those tools, a reference of comparison must be elaborated (Pesonen et al., 2000): it consists of the likely states of the pork value chain in the future (without the new tools being implemented). This implies to model all possible evolutions of the French pork value-chain so that we can eventually evaluate the impacts certain innovations might have on it: for that, we use prospective methods (Chaib et al., 2021). This goes beyond the scope of participatory modeling: indeed, it requires not only consulting and discussing with the stakeholders (Barré, 2000; Mermet, 2004), but collaborating with them to co-design a plausible future in order to co-decide what would be best for the value-chain. We are thus in a context of collaborative modeling as described in Basco-Carrera et al. (2017).

In project Sentinel we choose to use the French prospective Godet method in which scenarios are created based on the identification of key variables (Godet, 2008), i.e., variables of the system studied which both influence many other variables and are dependent from many variables, thus constituting instable points of the system. This is classically performed through collective face-to-face sessions with stakeholders. This method was however adapted in Chaib et al. (2021), due to the sanitary context, which required multiple individual remote sessions instead of collective face-to-face ones. In addition, it partly demanded the analysis of documents related to the subject. In consequence, by confronting all data sources, not only did we have different ontologies between stakeholders –which is avoided in collective sessions where a consensual vocabulary is used–, but those also differed from the ontologies of written documents.

In this paper, we explain how we construct ontologies manually in the adapted Godet method based on interviews and documents. However, doing so is very time consuming, and gaining time would be valuable. Plus, the final list of key variables has to be reconfirmed with the stakeholders by using the Delphi method (Chaib et al., 2021). We thought it would be complementary to test how the MyChoice multicriteria argumentation tool (Thomopoulos et al., 2020) can help alleviate the disadvantages of the adapted Godet method. It could maybe help in speeding up the process of constructing the variable ontologies. This can also ensure a complete and thorough analysis of what is being said in order to increase stakeholders' awareness of certain critical situations in agri-food value-chains. For those reasons we aim to explore the complementarities and redundancies of both methods.

The question addressed by the paper is whether prospective studies may have to gain by complementing the methodologies used (here Godet) with formal approaches from other disciplines, such as knowledge engineering (here MyChoice), which is usually not the case currently. The objective of the paper is to discuss why and how this could be performed. Its contribution is to highlight that equivalent outputs to Godet's can be obtained through the MyChoice method, concomitantly saving time. In “Section 2” we will first discuss the inputs and outputs of both methods. In “Section 3”, we present the steps followed in order to construct the ontology in both methods. Then, in “Section 4” we examine how the adapted Godet method is relevant to our study and how the MyChoice tool can possibly help in analyzing and confirming our results. Throughout the paper, examples of what is obtained in our study on the French pork value-chain are given.

## 2. Inputs and outputs of the modeling process

The main goal of a collaborative knowledge representation model is to aid stakeholders so that they can make informed decisions. Constructing the model requires inputs which are then analyzed to provide outputs for decision support.

### 2.1. Inputs: Data from interviews and documents

Every decision making process relies on the analysis of information sourced. In our case, whether it is for the adapted Godet method or for MyChoice, information comes from different stakeholders of the value-chain as said before. It is mainly in the form of text since semi-directive interviews are conducted and then transcribed to ensure proper analysis later on. To the interviews we added documents since during the time of the study, remote work was a necessity considering the sanitary context. Each document read was considered as an interview done (Chaib et al., 2021). In total, for project Sentinel, 21 texts were analyzed, including 12 transcribed interviews and 9 documents (Le Teno, 2013; Delanoue and Roguet, 2014; INAPORC, 2019; Barberis et al., 2020; Hoste, 2020; Assemblée Nationale Commission Des Affaires Economiques, 2021; Hofmann, 2021a,b,c).

To ensure representativeness of inputs, the researchers seek gathering prospects from various domains of the agri-food sector, and avoid interviewing only persons with the same background, who are more likely to deliver the same viewpoints and variables. This is why, to cover all domains, we also added documents from literary reviews which provide factual and substantial information about the agri-food chain studied. Each document read is considered equivalent to an interview. To ensure the validity of the selection of interviewees (documents included), we checked the following. When split according to the partition of important stakeholders between seven categories by Mitchell et al. (1997), all the seven categories of stakeholders are addressed.

The vocabularies and the language used in the transcriptions stem from a natural discussion with the stakeholders. Each stakeholder has a different way of seeing things, analyzing and interpreting them, thus the vocabulary used from one interview to another may change even though the main idea remains the same. In addition to there being varieties between the interviews, the vocabularies also vary in the documents. This can be explained by the fact that authors of documents have time to proof-read and homogenize their words and sentences, especially those aiming to reflect a single idea, whereas stakeholders at most have a few minutes to put clear words on the idea they want to pass on. And so the question raised is: How do we treat a rather large sample of words and phrases in order to extract a limited sample of ideas?

The ultimate aim being constructing scenarios of the possible evolution of the pork value-chain, the method chosen is the French prospective collaborative Godet method as described in Godet (2008). It has the particularity of creating scenarios no stakeholder has thought of which makes the discussion and the results more interesting. In this method the problem of homogenizing ontologies is inexistent since stakeholders themselves meet and establish consensus on the main ideas to keep in mind. However, a harder option was forcibly developed because of the COVID-19 pandemic. This leads us to the following crucial topic in our paper: the outputs.

### 2.2. Outputs

The outputs obtained following the interviews are double: on one hand we have outputs by using the adapted Godet method, and on another hand, we have the outputs obtained using the MyChoice tool for multicriteria argumentation since we think this method can help in constructing the ontologies needed. Indeed, it provides a standardized data structure to describe the opinions collected from the interviews/documents; moreover, it allows capitalizing the vocabularies built from the first interviews/documents and suggesting items for the next ones. Both methods were initially created with different objectives in mind: the Godet method aims to identify key variables which are used for the creation of scenarios, whereas the MyChoice tool originally serves to pinpoint what may be the strengths and weaknesses of the value-chain.

#### 2.2.1. Outputs of the adapted Godet method

**Figure 1:**
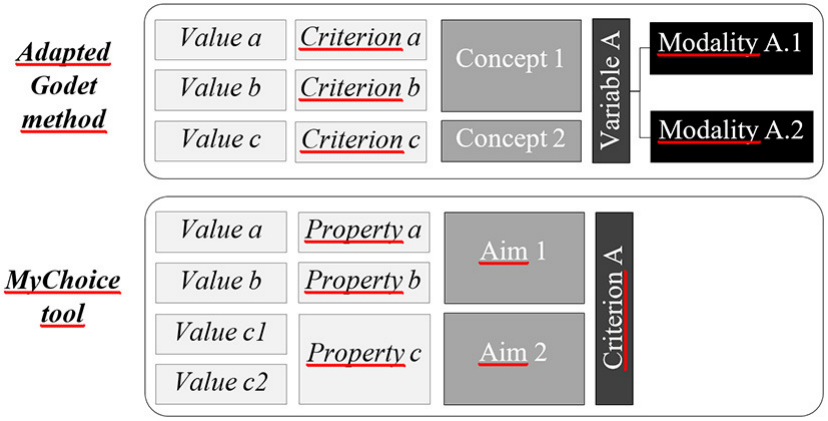
Nomenclatures of the adapted Godet method and the *MyChoice* tool.

The adapted Godet method first consists of extracting criteria referred to by the interviewees or documents to analyze the agri-food system development and perspectives. Similar criteria are then manually grouped into concepts by following an ontology matching procedure (Thomopoulos et al., 2013): basically, words or phrases which are synonyms or refer to the same idea are grouped. Concepts referring to the same global notion or theme are then grouped into variables. Each variable can take one or more value called modality (Figure 1).

For example, ≪ labor cost ≫ and ≪ need for investments ≫ are concepts of the variable ≪ production costs ≫ which can either take the modality ≪ production costs mastered ≫ or the modality ≪ fluctuating production costs ≫.

**Figure 2:**
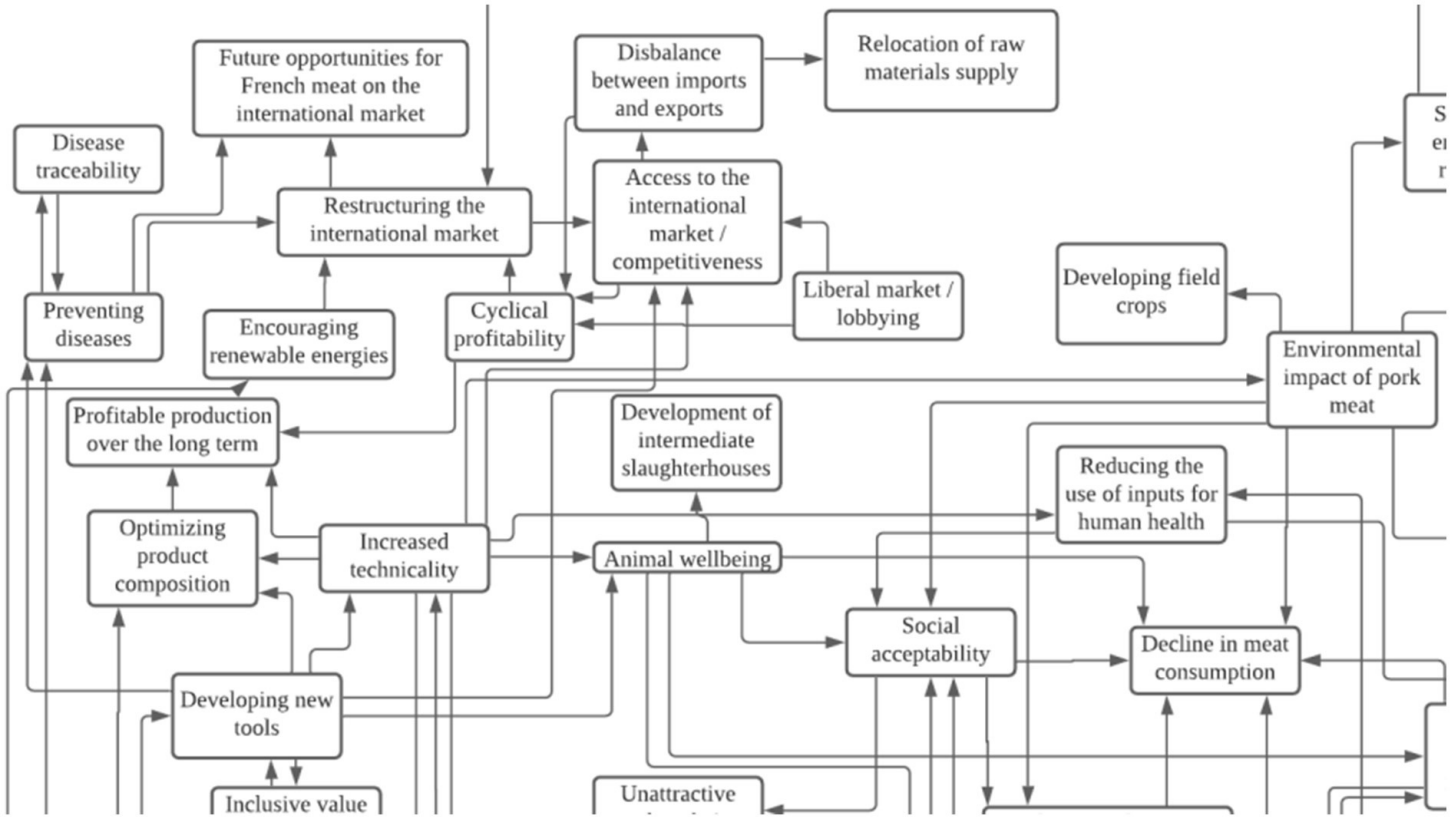
Extract of a mindmap showing relations between concepts identified in an interview.

Depending on the explanations given during the interviews or in the documents, a concept can either be found in only one variable (which is the case for most of them) or in two variables or more. It is important to note that the variables and their modalities are identified in the list of concepts. The identified concepts are linked to each other by influence and dependence relations identified in the transcriptions and documents and represented in mindmaps (Figure 2). It is those relations which eventually help us identify key variables (Chaib et al., 2021).

The outputs obtained then have to be confirmed by the stakeholders interviewed: a Delphi questionnaire listing all identified variables is sent to them so that they can choose up to 5 important variables in the 12 listed —which include the variables proposed by themselves—, in order to discriminate the most consensually important ones.

#### 2.2.2. Outputs of the *MyChoice* tool

When using the *MyChoice* tool, we obtain a list of properties. Those properties are similar to what we call criteria in the adapted Godet method. Each property is attributed to an aim (which resemble the concepts of the adapted Godet method) and the aims are grouped into what is called criteria in the *MyChoice* tool but really is the variables of the adapted Godet method. The parallel between the denominations of each method is shown in Figure 1.

There are however two main differences to note between Godet and *MyChoice* when it comes to the properties and the aims. The first one is that in *MyChoice*, a property can take several values but is still considered as a single property, whereas in the adapted Godet method we would consider that there are as many criteria as values a property can take. The second difference is that each aim can only be attributed to one single criterion, when in the adapted Godet method, a concept can be attributed to one, two or more variables.

In addition to identifying criteria, aims and properties, the *MyChoice* tool helps quantify the attitude of a stakeholder concerning the alternative chosen (Thomopoulos et al., 2020). For project Sentinel, since our aim is to anticipate future evolutions of the pork value-chain, the alternative chosen is ‘pursuing business as usual'. The attitude –also called ‘degree of acceptability of the alternative'– is a value between 0 and 1. It reflects to what extent pursuing business as usual meets the aims a stakholder expressed (Thomopoulos et al., 2020). *MyChoice* can either give the global attitude or a stakeholder's specific attitude toward a single criterion or aim.

The following section shows how we go from the inputs to the outputs whether using the adapted Godet method or the multicriteria argumentation tool *MyChoice*.

## 3. From inputs to outputs, the ontology-building steps

**Figure 3:**
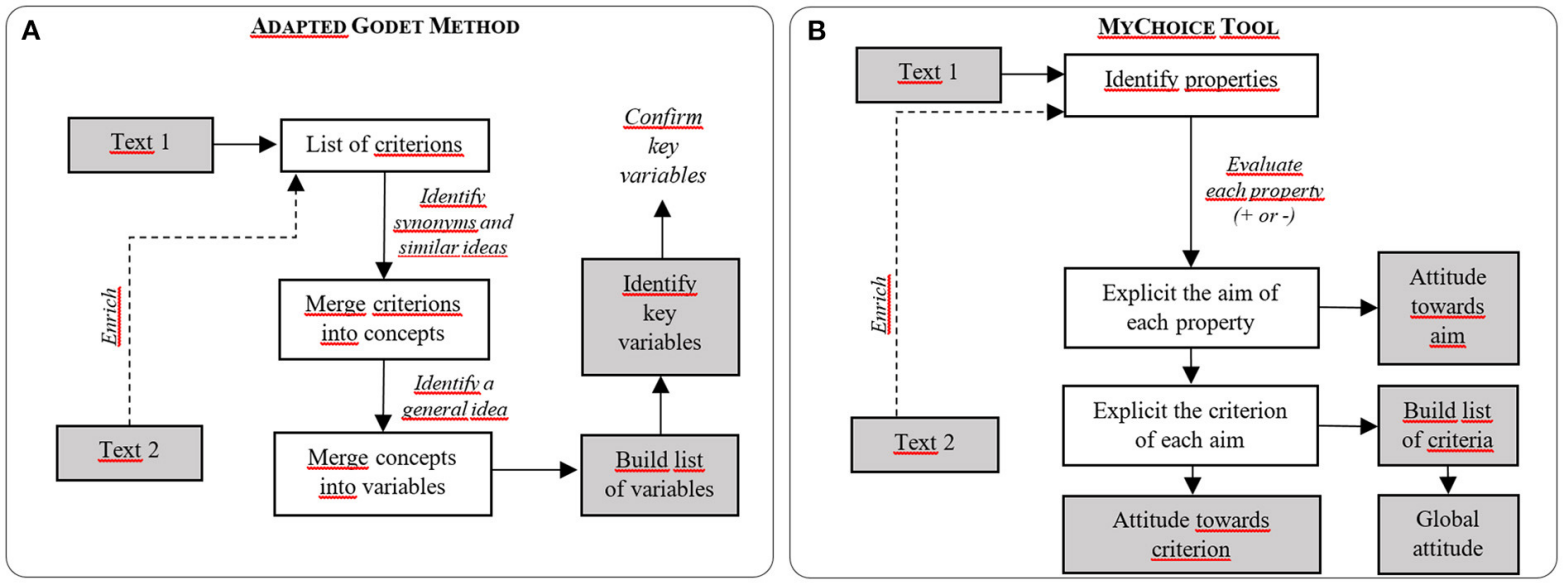
Steps of building an ontology in the adapted Godet method **(A)** and the *MyChoice* tool **(B)** (inputs and outputs are in gray). “Text 1” refers to an interview/document analyzed, while “Text 2” refers to a following one, which enriches to the list of properties identified with new ones.

**Figure 4:**
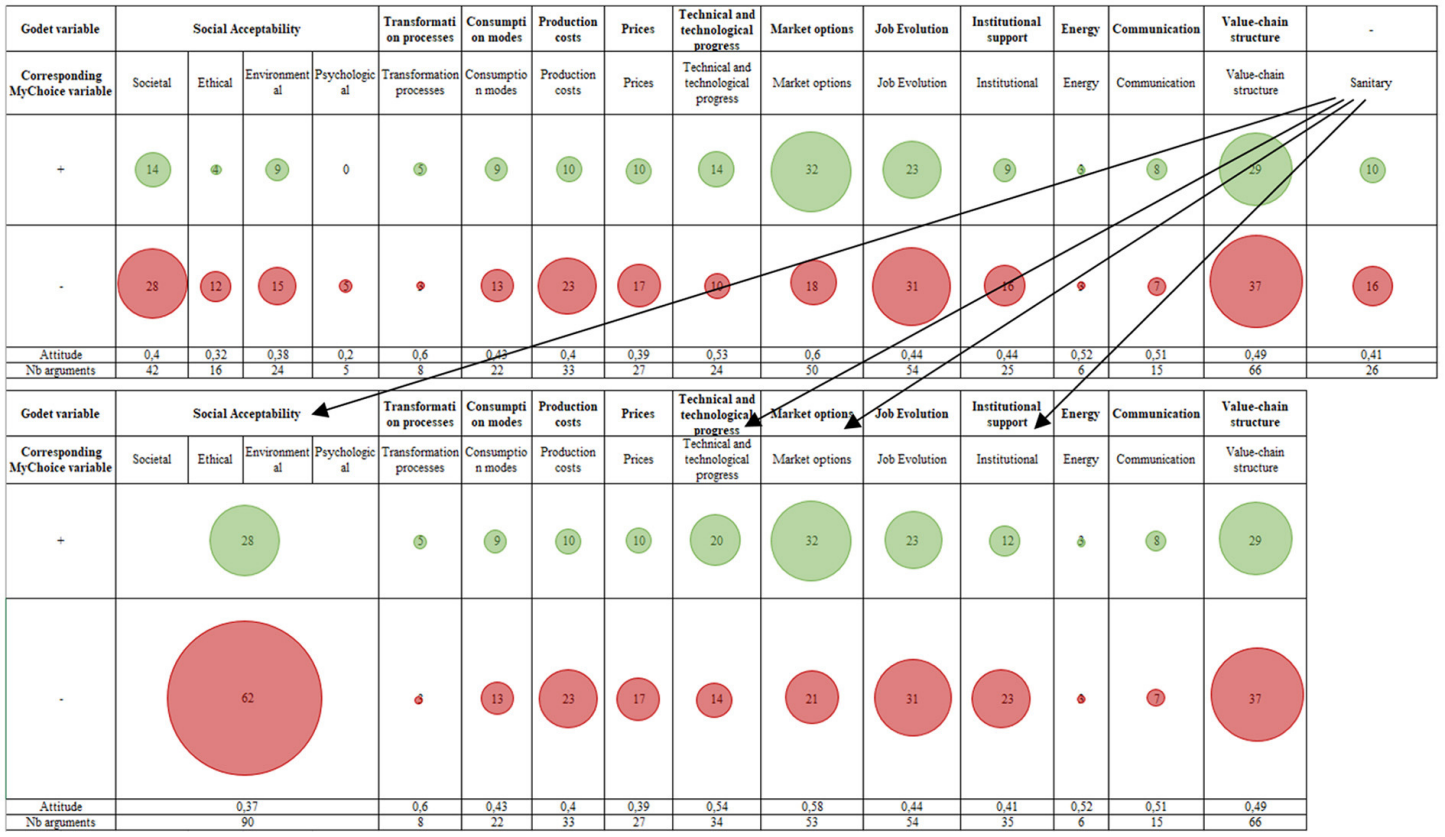
Identified variables in the adapted Godet method and the *MyChoice* tool. The top block details the quantities of positive and negative evaluations of the variables in the *MyChoice* tool. The bottom block details them for the adapted Godet method. Together with the explanations provided by the interviews, these evaluations are called “arguments”, counted in the last row of each block. The arrows express that information associated with the “Sanitary” variable in the *MyChoice* tool are dispatched in several variables (Social Acceptability, etc.) in the Godet method.

We saw in the previous section that the inputs for Godet and *MyChoice* are the same but the outputs are somewhat different. As for the processes followed: both methods are basically made of 3 main steps, as shown in Figure 3, allowing us to build lists of variables/ criteria, detailed in Figure 4.

In the adapted Godet method, it is best if all of the interviews are finished so that the process of merging similar criteria is a bit easier since it is done by hand. In the *MyChoice* tool, homogenizing the vocabularies used is a bit easier since the aims entered in the tool are automatically registered for future choices. Nevertheless, the process of attributing aims and criteria to properties is not automated.

## 4. Alignment of both models

The objective of the study is to build an ontology of variables –or criteria as they are called in the *MyChoice* tool– which influence the future of the value-chain and depend on it, so that we can eventually create the scenarios needed.

Using only the adapted Godet method, we were able to identify key variables and construct reference scenarios of the possible evolution of the value-chain. Nevertheless, the process of this method is very time consuming and complex as we said previously. That is why we thought about using the *MyChoice* tool for multicriteria argumentation, which allowed a gain of time approximately evaluated to a factor 5. In this section we compare the results obtained using both methods to see how they can be aligned when it comes to constructing the ontologies of variables.

To simplify the analysis, in the rest of the paper we adopt the nomenclatures of the adapted Godet method. Differences can be observed in the number of criteria, concepts and variables obtained using the adapted Godet method and the *MyChoice* tool, respectively: 626 versus 313 criteria, 169 versus 237 concepts, 12 versus 16 variables.

These differences can be explained as such:

— For the criteria: in the adapted Godet method, those are words or phrases extracted as is from the interviews and the documents. As for the criteria (argument) in *MyChoice*, they are a bit more general since a phrase from an interview is dissected into the property itself, a value attributed to it, and an evaluation (‘+' or ‘–' in Figure 4). In other words, in *MyChoice*, for a same denomination of a criterion, several values and evaluations can be attributed to it; they would be considered as different criteria in the adapted Godet method.
— For the concepts: in the adapted Godet method they are more general than the ones of *MyChoice*. A concept in Godet contains on average 4 criteria but can contain up to 24, whereas in *MyChoice* a concept contains 2 criteria on average and can have up to 12.
— As for the variables, they are more specific in the *MyChoice* database, however, some of them can be combined and it is possible to obtain 12 variables corresponding to those in the Godet method. This is more explicit in Figure 4.

The fact that *MyChoice* is an easy tool to use makes the process of homogenizing vocabularies and constructing ontologies of variables a bit easier: indeed, it is easier to avoid redundancies between criteria, because of the separation between values, evaluations and the denomination. We find ourselves with fewer denominations and a homogenized vocabulary. It is thus easier to group them into concepts manually. In addition, the fact that each concept can only belong to one aim forces us to be specific in the nomenclatures. The variables eventually obtained using the *MyChoice* tool correspond to the ones obtained by following the Godet adapted method.

*MyChoice* thus seems to be an adapted tool for the construction of an ontology of variables: it allows us to formalize and standardize vocabularies manually but still easily compared to the adapted Godet process. This allows a better exploitation of data in the future. The *MyChoice* tool however does not allow us to identify key variables, at least not for now; following the process of the adapted Godet method explicited previously and detailed in Chaib et al. (2021) seems inevitable. What the *MyChoice* tool could facilitate is the confirmation of key variables, especially since it is sometimes rather complicated to obtain responses of stakeholders when using the Delphi method.

## 5. Conclusion and future work

The objective of the paper was to explore whether and how prospective studies may have to gain from complementing the methodologies used (e.g., Godet) with formal approaches from other disciplines, such as knowledge engineering (e.g., MyChoice), which is usually not the case currently. The study highlighted that equivalent outputs to Godet's can be obtained through the MyChoice method, concomitantly improving the ease of structuring ontologies and saving time.

The work done in this paper emanated from the need to identify variables and especially key variables after interviewing stakeholders and reading documents about the possible evolution of the pork value-chain taken as an example in project Sentinel. The first option explored is an adaptation of the French prospective Godet method: it consists of extracting criteria from the texts, then manually assembling them into concepts which leads to an identification of general ideas or themes we call variables. The procedure being quite heavy, we searched for alternatives that could alleviate the disadvantages of this method while also saving us time. We decided to use *MyChoice* since it is an easy and accessible tool: it is useful tool for the construction of ontologies since it facilitates the process, and the results attained correspond to the ones obtained using the adapted Godet method. This tool would be even more adapted and useful if the process of combining criteria and concepts was fully automated.

## Data availability statement

The raw data supporting the conclusions of this article will be made available by the authors, without undue reservation.

## Ethics statement

Ethical review and approval was not required for the study involving human participants in accordance with the local legislation and institutional requirements. Written informed consent to participate in this study was not required from the participants in accordance with the national legislation and the institutional requirements.

## Author contributions

RC, CM, and RT contributed to conception and design of the study. RC wrote the first draft of the manuscript. All authors contributed to manuscript revision, read, and approved the submitted version.
